# Supplementary material for: A Large-Scale Candidate-Gene Association Mapping for Drought Tolerance and Agronomic Traits in Sugarcane
Source: Int J Mol Sci. 2023 Aug 15;24(16):12801. doi: 10.3390/ijms241612801 (PMC10454574; doi:10.3390/ijms241612801)

Figure S1. The accumulated monthly rainfall in two growing seasons of an irrigated (non-stressed, NS) and a rainfed (water stress, WS) field experiments

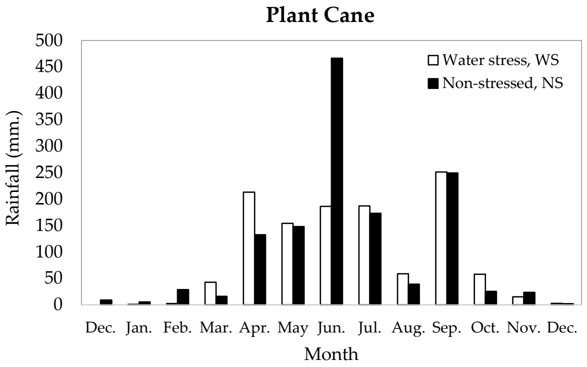

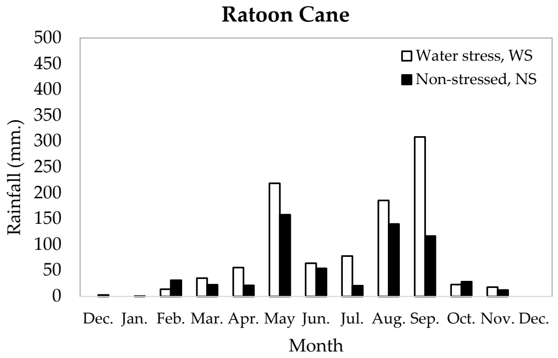

Figure S2. Chromosomal locations of 5,053 polymorphic SNP for population structure analysis. The 10 sugarcane chromosomes (Sh01-Sh10) are shown as vertical bars and each horizontal line on the bar represents SNP. The scale on left side represents physical map positions in Mbp.

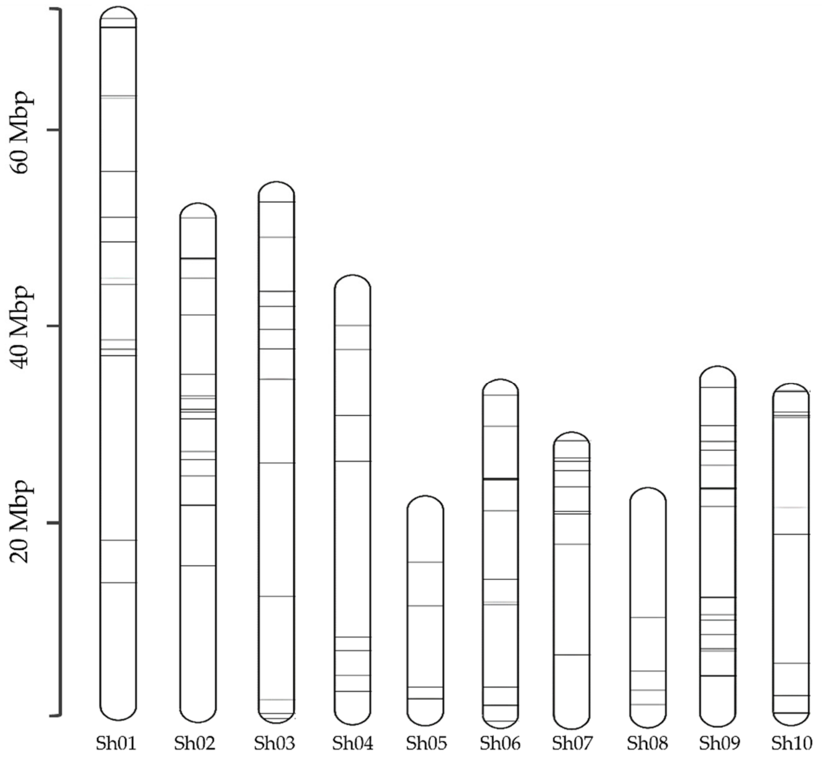

Figure S3. Distribution of 159 sugarcane accessions into 3 subpopulations obtained through the DAPC. The heat colors inside the columns indicate the set of accessions grouped in the corresponding subpopulation according to the membership probabilities (red =1 and white=0) based on DAPC and blue crosses represent the prior cluster provided to DAPC through k-mean method.

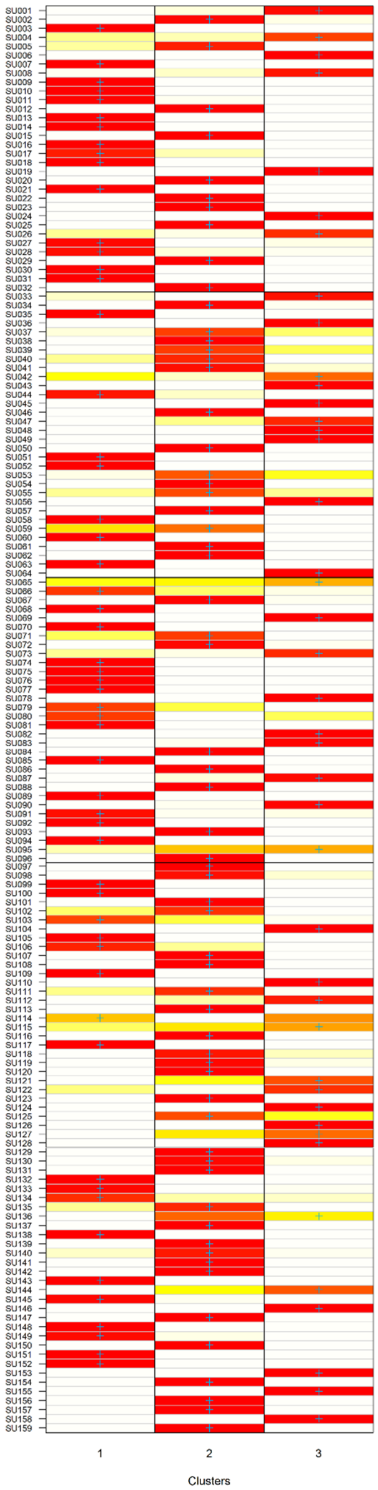

Figure S4. Manhattan plots and quantile-quantile (QQ) plots of MTAs for each agronomic trait of plant cane (P) and ratoon cane (R) evaluated under non-stressed (NS) and water-stressed (WS) conditions, in a panel of the 159 accessions using FarmCPU. The blue dotted line marks the threshold to declare a significant association based on Bonferroni-corrected significance threshold α=0.05 (p-value ≤ 4.26 x 10^{-6}). (a) cane length (CL), (b) cane diameter (CD), (c) internode length (IL), (d) number of millable canes (NMC), (e) single cane weight (SCW), (f) cane yield (CY), (g) sugar yield (SY), (h) ratooning ability (RA), (i) brix (BR), (j) commercial cane sugar (CCS), (k) polarization (PO), (l) purity (PU) and (m) fiber (FB)

a

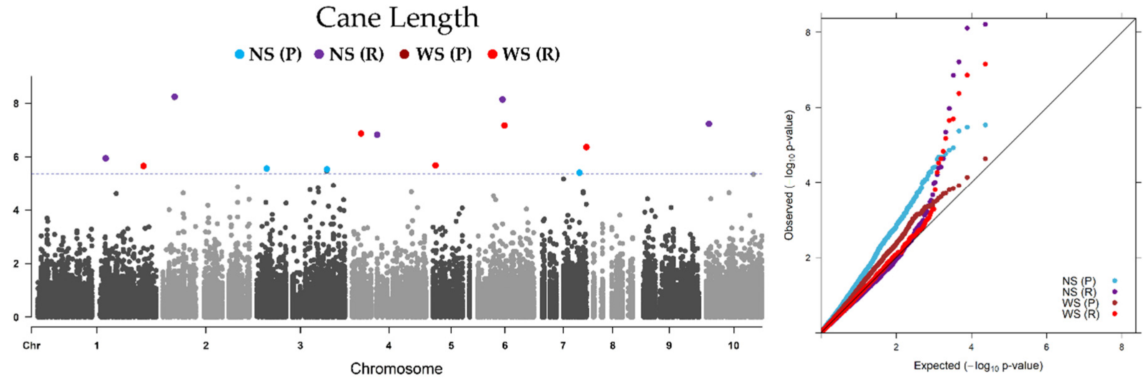

b

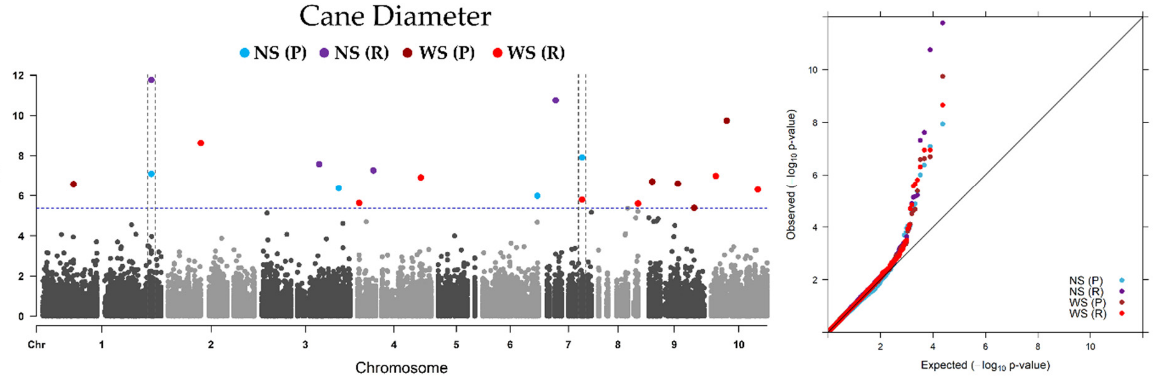

c

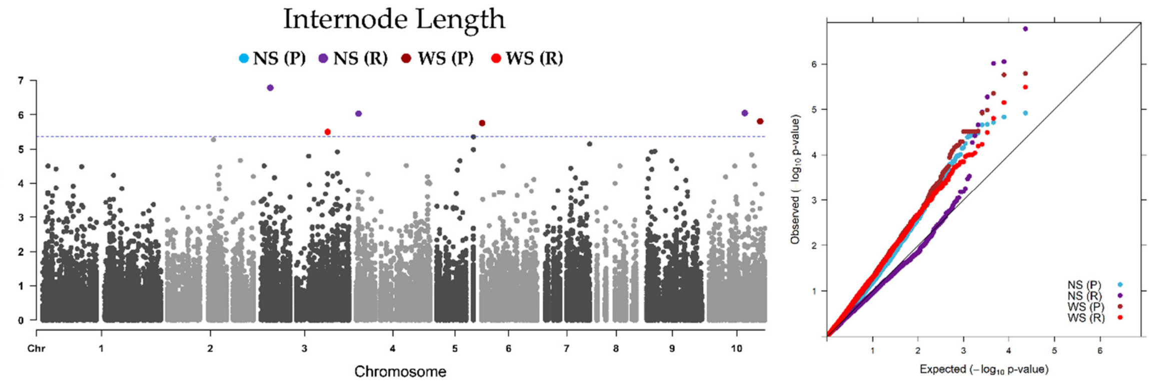

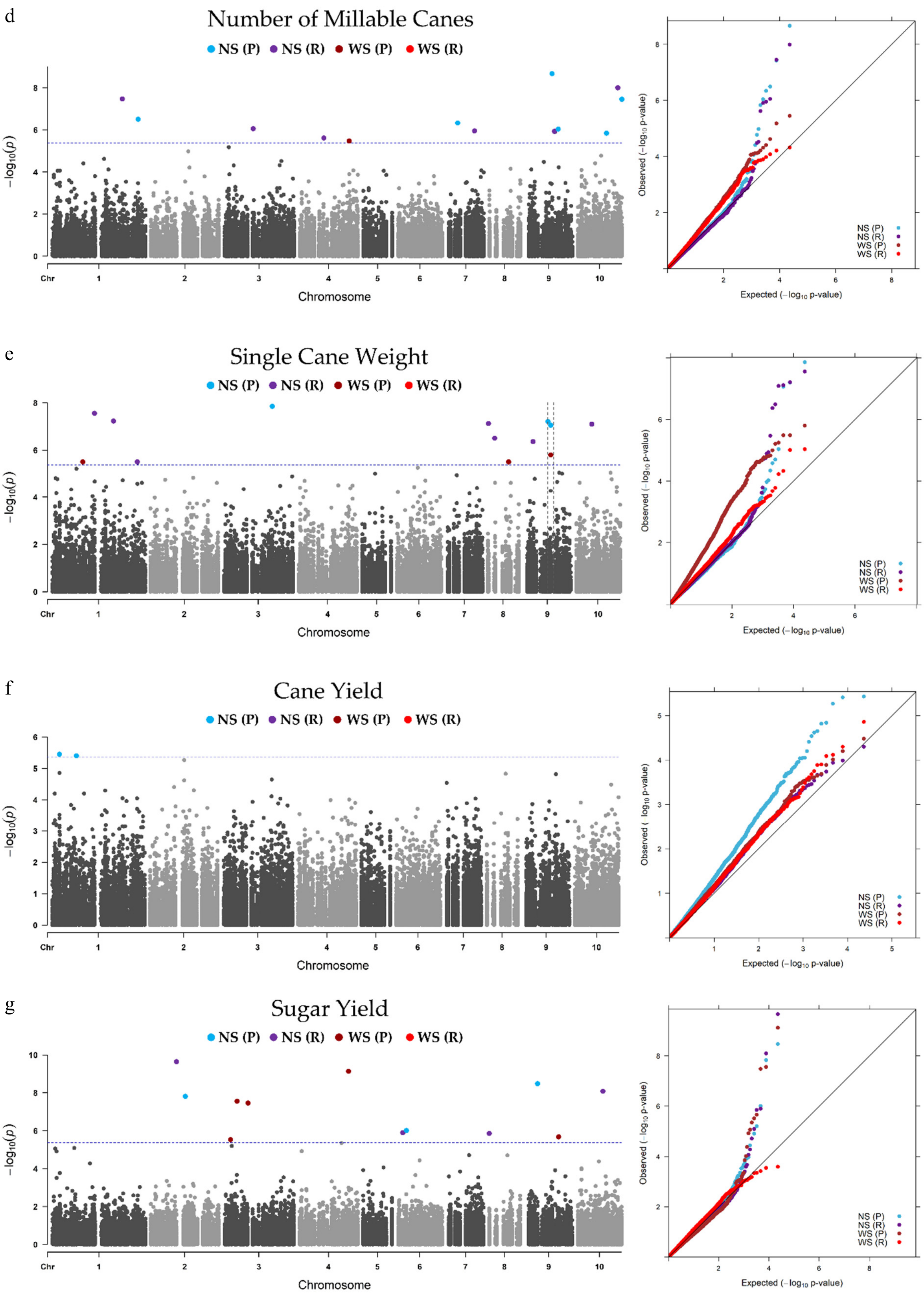

d
Number of Millable Canes
NS (P) NS (R) WS (P) WS (R)
−log10(p)
Chromosome
Observed (−log10 p-value)
Expected (−log10 p-value)
e
Single Cane Weight
NS (P) NS (R) WS (P) WS (R)
−log10(p)
Chromosome
Observed (−log10 p-value)
Expected (−log10 p-value)
f
Cane Yield
NS (P) NS (R) WS (P) WS (R)
−log10(p)
Chromosome
Expected (−log10 p-value)
g
Sugar Yield
NS (P) NS (R) WS (P) WS (R)
−log10(p)
Chromosome
Observed (−log10 p-value)
Expected (−log10 p-value)

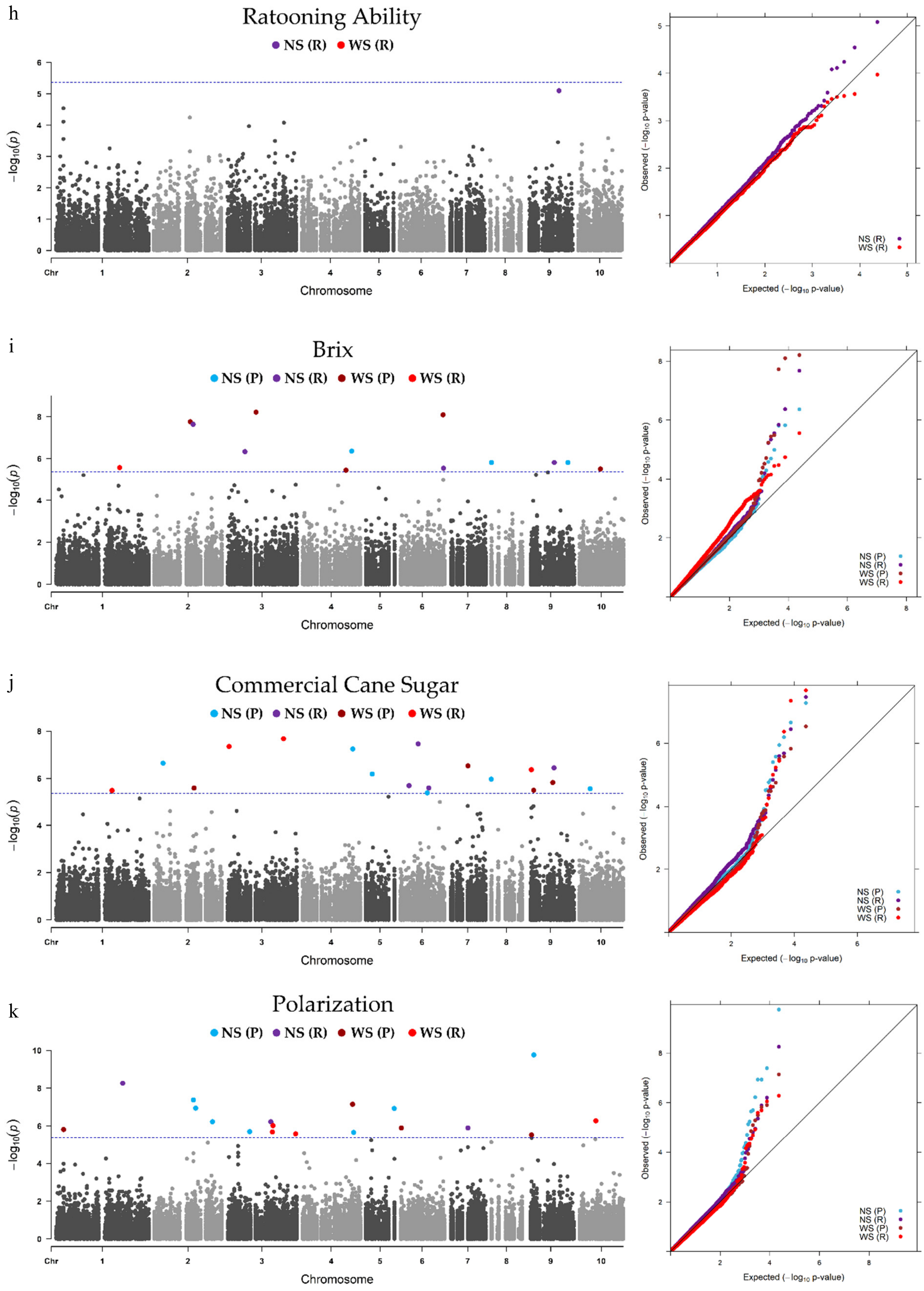

h
Ratooning Ability
NS (R)
WS (R)
i
Brix
NS (P)
NS (R)
WS (P)
WS (R)
j
Commercial Cane Sugar
NS (P)
NS (R)
WS (P)
WS (R)
k
Polarization
NS (P)
NS (R)
WS (P)
WS (R)
−log10(p)
Chr
Chromosome
Observed (−log10 p-value)
Expected (−log10 p-value)

l

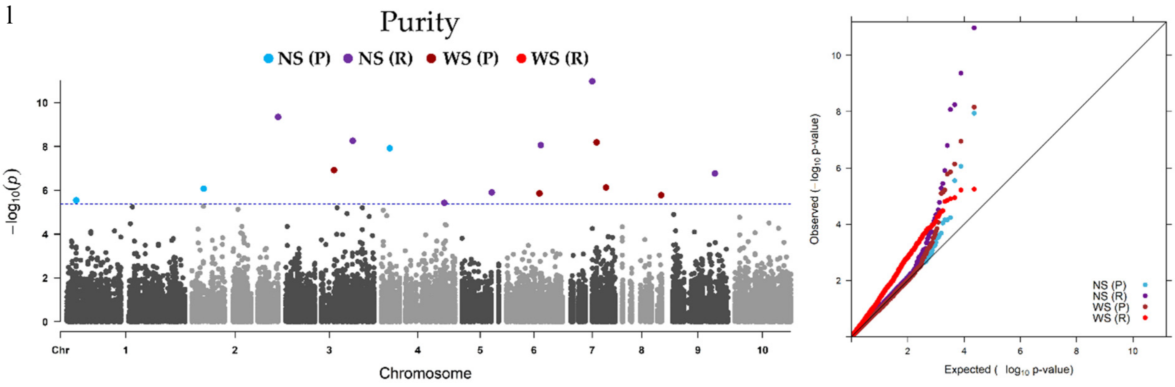
Purity
NS (P)
NS (R)
WS (P)
WS (R)
−log10(p)
Chr
Chromosome
Observed (−log10 p-value)
Expected (log10 p-value)

m

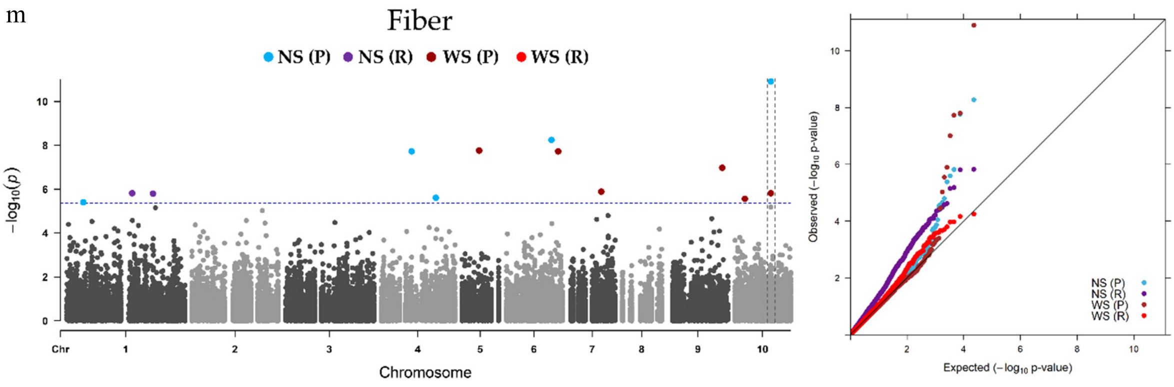
Fiber
NS (P)
NS (R)
WS (P)
WS (R)
−log10(p)
Chr
Chromosome
Observed (−log10 p-value)
Expected (−log10 p-value)

Figure S5. Manhattan plots and quantile-quantile (QQ) plots of MTAs for the drought-tolerant coefficient (DC) and the membership function value of drought tolerance (MFVD), in a panel of the 159 accessions using FarmCPU. The blue dotted line marks the threshold to declare a significant association based on Bonferroni-corrected significance threshold α=0.05 (p-value ≤ 4.26 x 10^{-6}). The drought-tolerant coefficient of (a) internode length (DC_{IL}), (b) single cane weight (DC_{SCW}), (c) cane yield (DC_{CY}), (d) sugar yield (DC_{SY}), and (e) the MFVD.

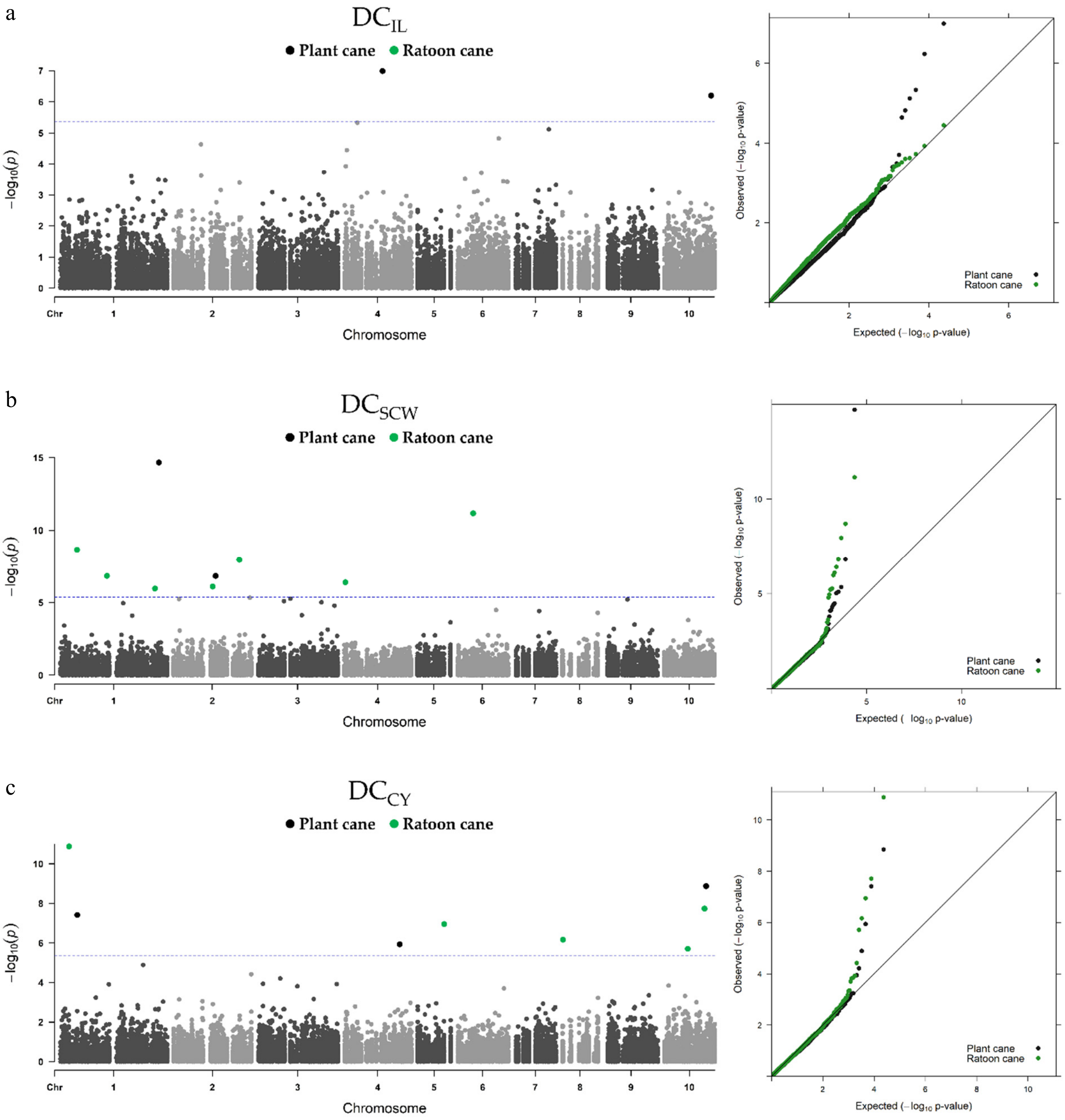

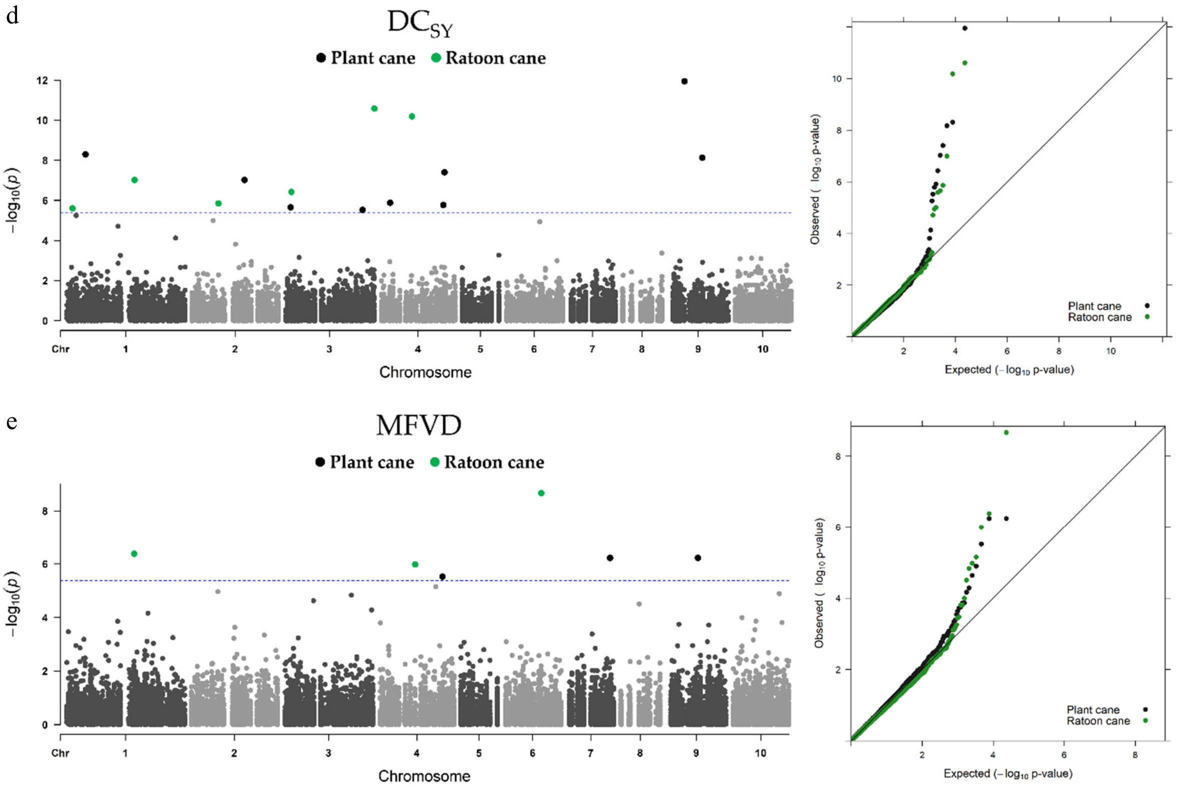

d
DCSY
Plant cane
Ratoon cane
−log10(p)
Chr
1
2
3
4
5
6
7
8
9
10
Chromosome
Observed (−log10 p-value)
Expected (−log10 p-value)
Plant cane
Ratoon cane
e
MFVD
Plant cane
Ratoon cane
−log10(p)
Chromosome
Observed (−log10 p-value)
Expected (−log10 p-value)
Plant cane
Ratoon cane

Figure S6. The distribution of yield-related traits and sugar-related traits of plant cane and ratoon cane under non-stressed (NS) and water-stressed (WS) conditions, in a panel of the 159 accessions. Each trait was tested for normality using Shapiro-Wilks (p < 0.05).

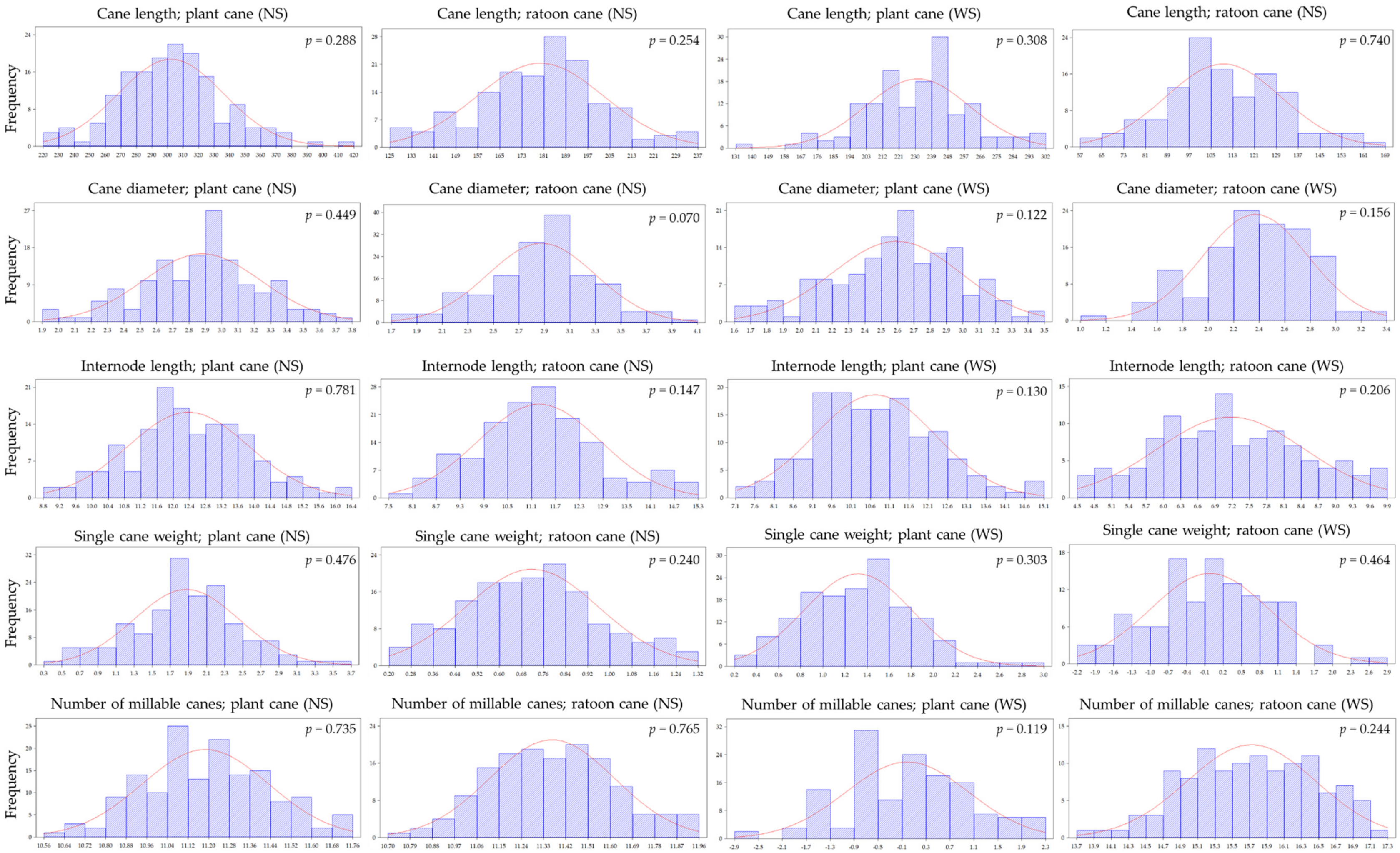

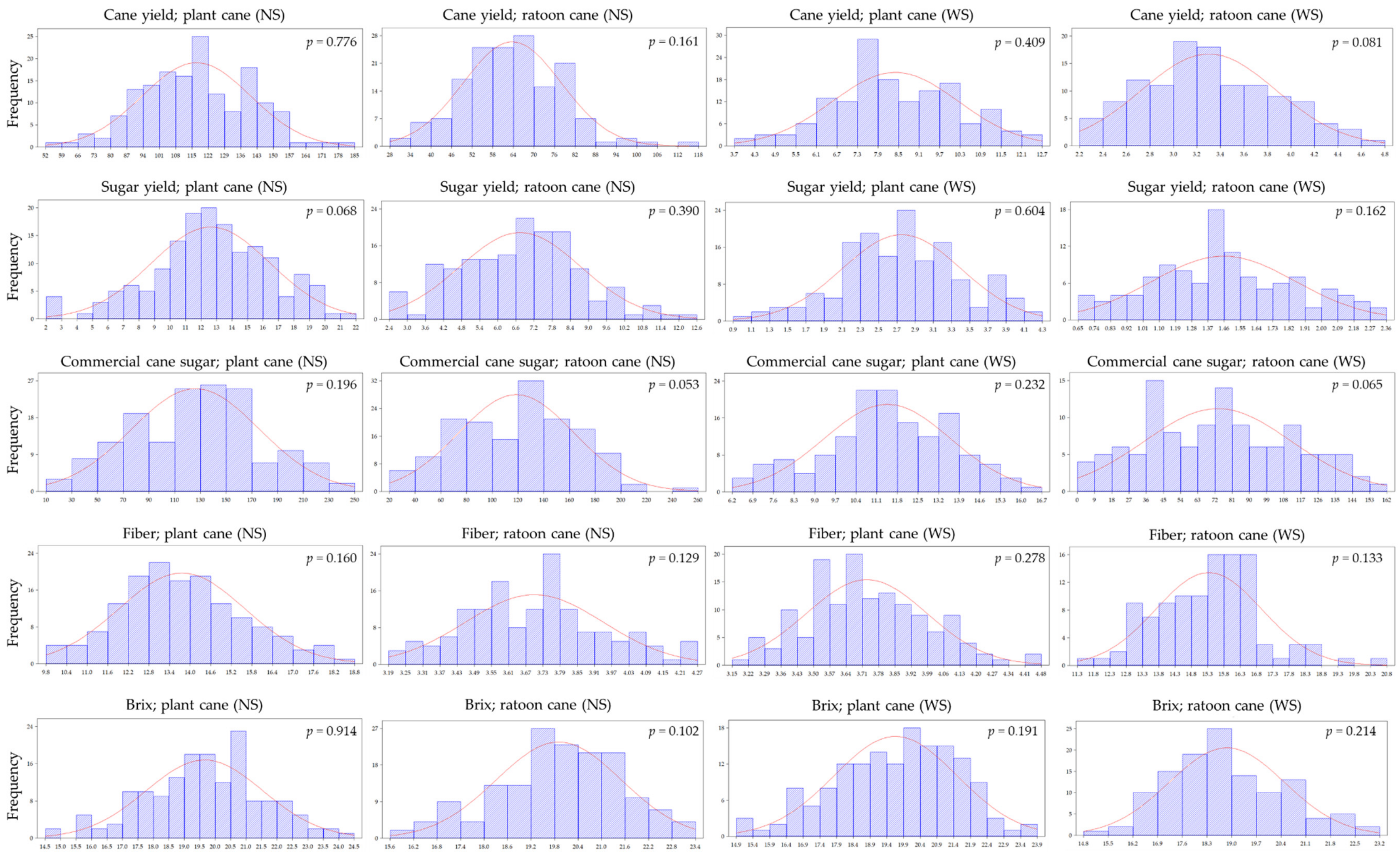
Cane yield; plant cane (NS)
p = 0.776
Cane yield; ratoon cane (NS)
p = 0.161
Cane yield; plant cane (WS)
p = 0.409
Cane yield; ratoon cane (WS)
p = 0.081
Sugar yield; plant cane (NS)
p = 0.068
Sugar yield; ratoon cane (NS)
p = 0.390
Sugar yield; plant cane (WS)
p = 0.604
Sugar yield; ratoon cane (WS)
p = 0.162
Commercial cane sugar; plant cane (NS)
p = 0.196
Commercial cane sugar; ratoon cane (NS)
p = 0.053
Commercial cane sugar; plant cane (WS)
p = 0.232
Commercial cane sugar; ratoon cane (WS)
p = 0.065
Fiber; plant cane (NS)
p = 0.160
Fiber; ratoon cane (NS)
p = 0.129
Fiber; plant cane (WS)
p = 0.278
Fiber; ratoon cane (WS)
p = 0.133
Brix; plant cane (NS)
p = 0.914
Brix; ratoon cane (NS)
p = 0.102
Brix; plant cane (WS)
p = 0.191
Brix; ratoon cane (WS)
p = 0.214
Frequency

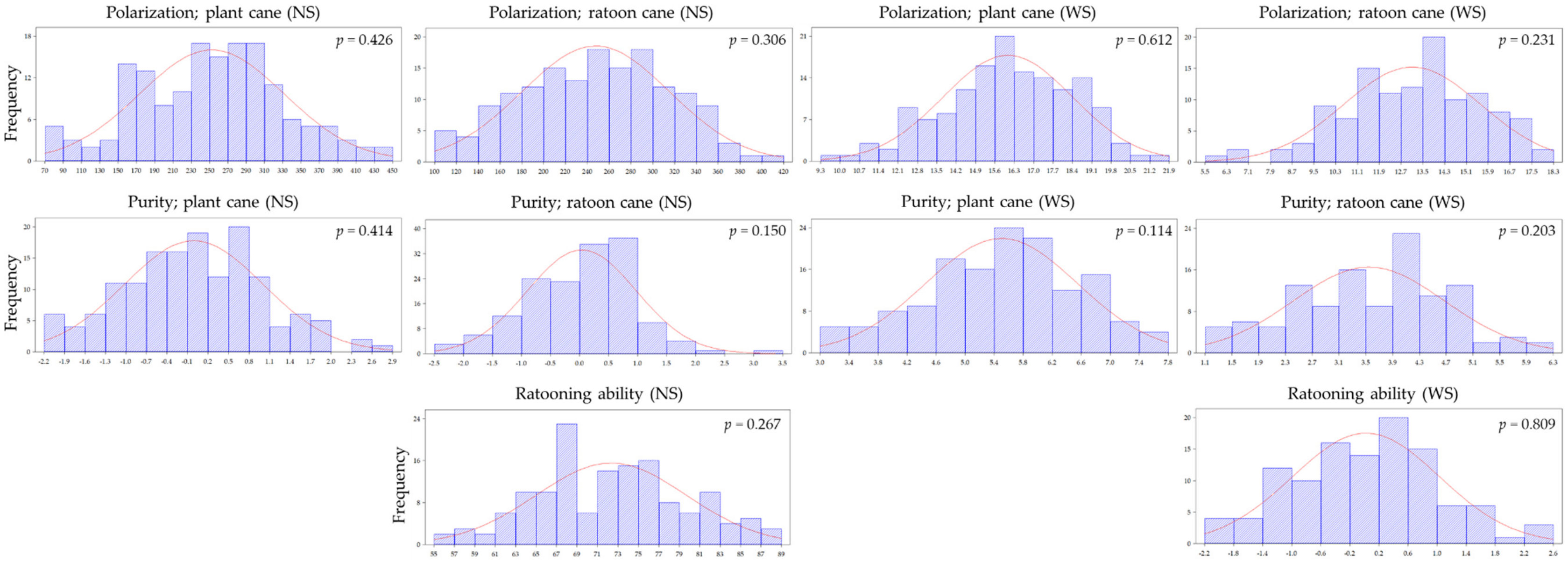
Polarization; plant cane (NS)
p = 0.426
Polarization; ratoon cane (NS)
p = 0.306
Polarization; plant cane (WS)
p = 0.612
Polarization; ratoon cane (WS)
p = 0.231
Purity; plant cane (NS)
p = 0.414
Purity; ratoon cane (NS)
p = 0.150
Purity; plant cane (WS)
p = 0.114
Purity; ratoon cane (WS)
p = 0.203
Ratooning ability (NS)
p = 0.267
Ratooning ability (WS)
p = 0.809
Frequency

Figure S7. The distribution of the drought-tolerant coefficient (DC) and the membership function value of drought tolerance (MFVD), in a panel of the 159 accessions of plant cane and ratoon cane. Each trait was tested for normality using Shapiro-Wilks ($p < 0.05$). DC = Drought-tolerant coefficient of each yield traits; MFVD = the average value of the member-ship function of all the traits

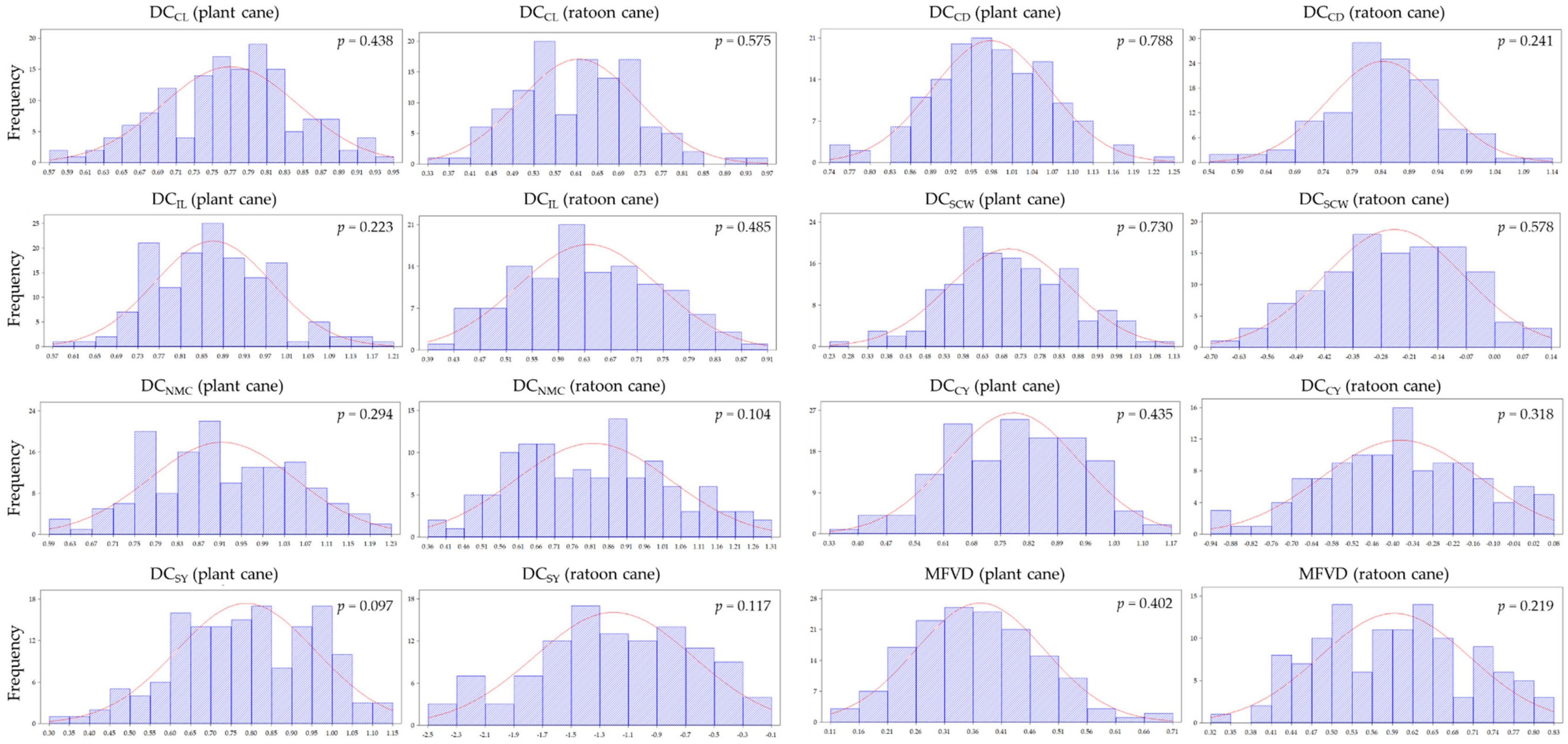

Supplement: Supplementary file 1 [file ijms-24-12801-s001.zip › Supplementary Materials Figure.pdf]
