# Supplementary material for: A Large-Scale Candidate-Gene Association Mapping for Drought Tolerance and Agronomic Traits in Sugarcane
Source: Int J Mol Sci. 2023 Aug 15;24(16):12801. doi: 10.3390/ijms241612801 (PMC10454574; doi:10.3390/ijms241612801)
Supplement: Supplementary file 1 [file ijms-24-12801-s001.zip › Supplementary Materials Table.pdf]

**Table S1.** Details of 159 sugarcane accessions used in the present study

| ID | Accession | Country of origin | Member possibility | | | Subpopulation |
|---|---|---|---|---|---|---|
| | | | Cluster 1 | Cluster 2 | Cluster 3 | |
| SU001 | MPT02-458 | Thai breeding line | 0 | 0.03 | 0.97 | 3 |
| SU002 | F152 | Taiwan | 0 | 0.97 | 0.02 | 2 |
| SU003 | LF48-8077 | Fiji | 1 | 0 | 0 | 1 |
| SU004 | MPT04-169 | Thai breeding line | 0.12 | 0.07 | 0.81 | 3 |
| SU005 | MPT07-152 | Thai breeding line | 0.09 | 0.91 | 0 | 2 |
| SU006 | KKU99-06 | Thailand | 0 | 0 | 1 | 3 |
| SU007 | ROC7 | Taiwan | 1 | 0 | 0 | 1 |
| SU008 | MPT04-467 | Thai breeding line | 0.01 | 0.05 | 0.94 | 3 |
| SU009 | Q208 | Australia | 1 | 0 | 0 | 1 |
| SU010 | F148 | Taiwan | 1 | 0 | 0 | 1 |
| SU011 | Co290 | India | 0.99 | 0.01 | 0 | 1 |
| SU012 | CP45-150 | USA | 0 | 1 | 0 | 2 |
| SU013 | M124/59 | Mauritius | 0.99 | 0 | 0.01 | 1 |
| SU014 | Q100 | Australia | 1 | 0 | 0 | 1 |
| SU015 | K95-84 | Thailand | 0 | 1 | 0 | 2 |
| SU016 | B4744 | Barbados | 1 | 0 | 0 | 1 |
| SU017 | B76718 | Barbados | 0.92 | 0.08 | 0 | 1 |
| SU018 | Co1287 | India | 1 | 0 | 0 | 1 |
| SU019 | UT1 | Thailand | 0 | 0 | 1 | 3 |
| SU020 | MPT06-26 | Thai breeding line | 0 | 1 | 0 | 2 |
| SU021 | LF79-2964 | Fiji | 1 | 0 | 0 | 1 |
| SU022 | LK92-11 | Thailand | 0 | 1 | 0 | 2 |
| SU023 | MPT14-1-892 | Thai breeding line | 0 | 1 | 0 | 2 |
| SU024 | MPT12-1782 | Thai breeding line | 0 | 0 | 1 | 3 |
| SU025 | Biotec 3 | Thailand | 0 | 1 | 0 | 2 |
| SU026 | UT6 | Thailand | 0.11 | 0.03 | 0.86 | 3 |
| SU027 | NCo310 | South Africa | 0.97 | 0 | 0.03 | 1 |
| SU028 | Mossman | Australia | 0.96 | 0.03 | 0.01 | 1 |
| SU029 | B41721 | Barbados | 0 | 1 | 0 | 2 |
| SU030 | N50-211 | South Africa | 0.99 | 0 | 0.01 | 1 |
| SU031 | IAC51-205 | Brazil | 1 | 0 | 0 | 1 |
| SU032 | BO24 | India | 0 | 1 | 0 | 2 |
| SU033 | MPT06-171 | Thai breeding line | 0.05 | 0 | 0.95 | 3 |
| SU034 | MPT14-1-504 | Thai breeding line | 0 | 0.99 | 0.01 | 2 |
| SU035 | Pindar | Australia | 1 | 0 | 0 | 1 |
| SU036 | MPT07-71 | Thai breeding line | 0 | 0 | 1 | 3 |
| SU037 | MPT06-144 | Thai breeding line | 0.04 | 0.82 | 0.15 | 2 |
| SU038 | MPT14-5-239 | Thai breeding line | 0 | 1 | 0 | 2 |
| SU039 | Fiji105 | Fiji | 0.01 | 0.83 | 0.16 | 2 |
| SU040 | Galoa | Unknown | 0.11 | 0.89 | 0 | 2 |
| SU041 | Kps01-25 | Thailand | 0 | 0.95 | 0.05 | 2 |
| SU042 | MPT09-296 | Thai breeding line | 0.25 | 0.05 | 0.7 | 3 |
| SU043 | KKU99-02 | Thailand | 0 | 0 | 1 | 3 |
| SU044 | ROC1 | Taiwan | 0.93 | 0.06 | 0.01 | 1 |
| SU045 | F170 | Taiwan | 0 | 0 | 1 | 3 |
| SU046 | MPT14-6-63 | Thai breeding line | 0 | 1 | 0 | 2 |
| SU047 | MPT06-367 | Thai breeding line | 0 | 0.12 | 0.88 | 3 |
| SU048 | Co331 | India | 0 | 0 | 1 | 3 |
| SU049 | CP75-324 | USA | 0 | 0 | 1 | 3 |
| SU050 | M147/158 | Mauritius | 0 | 1 | 0 | 2 |
| SU051 | Q83 | Australia | 1 | 0 | 0 | 1 |
| SU052 | Yasawa | Fiji | 1 | 0 | 0 | 1 |
| SU053 | MPT06-362 | Thai breeding line | 0.02 | 0.75 | 0.23 | 2 |
| SU054 | MPT14-1-180 | Thai breeding line | 0 | 1 | 0 | 2 |
| SU055 | MPT04-303 | Thai breeding line | 0.11 | 0.78 | 0.11 | 2 |

| ID | Accession | Country of origin | Member possibility | | | Subpopulation |
|---|---|---|---|---|---|---|
| | | | Cluster 1 | Cluster 2 | Cluster 3 | |
| SU056 | K99-72 | Thailand | 0 | 0 | 1 | 3 |
| SU057 | BL4 | Sri Lanka | 0 | 1 | 0 | 2 |
| SU058 | 3-2-023L | Thailand | 1 | 0 | 0 | 1 |
| SU059 | MPT08-50 | Thai breeding line | 0.32 | 0.67 | 0.01 | 2 |
| SU060 | Q81 | Australia | 1 | 0 | 0 | 1 |
| SU061 | F153 | Taiwan | 0 | 1 | 0 | 2 |
| SU062 | CP72-120 | USA | 0 | 1 | 0 | 2 |
| SU063 | CN1 | Thailand | 1 | 0 | 0 | 1 |
| SU064 | SP80 | Thailand | 0 | 0 | 1 | 3 |
| SU065 | Kpk98-40 | Thailand | 0.23 | 0.28 | 0.49 | Admixture |
| SU066 | SP50 | Thailand | 0.83 | 0.15 | 0.02 | 1 |
| SU067 | Kps01-12 | Thailand | 0 | 0.98 | 0.02 | 2 |
| SU068 | PSA63 | Philippines | 1 | 0 | 0 | 1 |
| SU069 | F174 | Taiwan | 0 | 0 | 1 | 3 |
| SU070 | Mackay | Australia | 1 | 0 | 0 | 1 |
| SU071 | Q146 | Australia | 0.16 | 0.83 | 0.01 | 2 |
| SU072 | F177 | Taiwan | 0.01 | 0.98 | 0.01 | 2 |
| SU073 | MPT02-669 | Thai breeding line | 0.1 | 0.02 | 0.88 | 3 |
| SU074 | Yuan Jiang | Unknown | 1 | 0 | 0 | 1 |
| SU075 | MY55-14 | Cuba | 1 | 0 | 0 | 1 |
| SU076 | M13/58 | Mauritius | 1 | 0 | 0 | 1 |
| SU077 | Q47 | Australia | 1 | 0 | 0 | 1 |
| SU078 | MPT05-187 | Thai breeding line | 0 | 0 | 1 | 3 |
| SU079 | MP1 | Thai breeding line | 0.81 | 0.19 | 0 | 1 |
| SU080 | IRK67-1 | Unknown | 0.82 | 0.01 | 0.17 | 1 |
| SU081 | F140 | Taiwan | 1 | 0 | 0 | 1 |
| SU082 | MPT99-1444 | Thai breeding line | 0 | 0 | 1 | 3 |
| SU083 | K93-219 | Thailand | 0 | 0.02 | 0.98 | 3 |
| SU084 | KK3 | Thailand | 0 | 1 | 0 | 2 |
| SU085 | Q77 | Australia | 1 | 0 | 0 | 1 |
| SU086 | MPT06-169 | Thai breeding line | 0 | 1 | 0 | 2 |
| SU087 | MPT09-204 | Thai breeding line | 0 | 0.03 | 0.97 | 3 |
| SU088 | PR3067 | USA | 0 | 1 | 0 | 2 |
| SU089 | Eros | Australia | 1 | 0 | 0 | 1 |
| SU090 | MPT04-55 | Thai breeding line | 0.01 | 0.01 | 0.98 | 3 |
| SU091 | Tuc74-6 | Argentina | 0.96 | 0.02 | 0.03 | 1 |
| SU092 | BO14 | India | 1 | 0 | 0 | 1 |
| SU093 | MPT14-1-902 | Thai breeding line | 0 | 1 | 0 | 2 |
| SU094 | LF76-2300 | Fiji | 1 | 0 | 0 | 1 |
| SU095 | UT5 | Thailand | 0.08 | 0.43 | 0.5 | Admixture |
| SU096 | MPT14-1-857 | Thai breeding line | 0 | 1 | 0 | 2 |
| SU097 | DB7160 | Barbados | 0 | 1 | 0 | 2 |
| SU098 | LF78-960 | Fiji | 0 | 0.95 | 0.04 | 2 |
| SU099 | MP2 | Thai breeding line | 1 | 0 | 0 | 1 |
| SU100 | R397 | Reunion | 1 | 0 | 0 | 1 |
| SU101 | Biotec 5 | Thailand | 0 | 1 | 0 | 2 |
| SU102 | MPT14-2-662 | Thai breeding line | 0.15 | 0.85 | 0 | 2 |
| SU103 | Phil66-07 | Philippines | 0.8 | 0.19 | 0.02 | 1 |
| SU104 | CP63-306 | USA | 0 | 0 | 1 | 3 |
| SU105 | Mana | Fiji | 1 | 0 | 0 | 1 |
| SU106 | GT29 | China | 0.89 | 0.11 | 0 | 1 |
| SU107 | Biotec 2 | Thailand | 0 | 1 | 0 | 2 |
| SU108 | Biotec 1 | Thailand | 0 | 1 | 0 | 2 |
| SU109 | Q117 | Australia | 1 | 0 | 0 | 1 |
| SU110 | MPT07-1 | Thai breeding line | 0 | 0 | 1 | 3 |
| SU111 | Tuc72-5 | Argentina | 0.11 | 0.87 | 0.02 | 2 |
| SU112 | Kwt3 | China | 0 | 0.08 | 0.92 | 3 |

| ID | Accession | Country of origin | Member possibility | | | Subpopulation |
|---|---|---|---|---|---|---|
| | | | Cluster 1 | Cluster 2 | Cluster 3 | |
| SU113 | K92-80 | Thailand | 0 | 1 | 0 | 2 |
| SU114 | MPT06-166 | Thai breeding line | 0.42 | 0.01 | 0.57 | Admixture |
| SU115 | MPT08-191 | Thai breeding line | 0.16 | 0.32 | 0.52 | Admixture |
| SU116 | Biotec 4 | Thailand | 0 | 1 | 0 | 2 |
| SU117 | Ebene1/37 | Mauritius | 1 | 0 | 0 | 1 |
| SU118 | UT8 | Thailand | 0 | 0.94 | 0.06 | 2 |
| SU119 | MPT02-665 | Thai breeding line | 0 | 0.98 | 0.02 | 2 |
| SU120 | PL310 | Unknown | 0 | 1 | 0 | 2 |
| SU121 | MPT11-82 | Thai breeding line | 0 | 0.24 | 0.76 | 3 |
| SU122 | MPT08-3 | Thai breeding line | 0.13 | 0.01 | 0.86 | 3 |
| SU123 | MPT12-141 | Thai breeding line | 0 | 1 | 0 | 2 |
| SU124 | UT3 | Thailand | 0 | 0 | 1 | 3 |
| SU125 | MPT09-118 | Thai breeding line | 0 | 0.78 | 0.22 | 2 |
| SU126 | CP76-340 | USA | 0 | 0 | 1 | 3 |
| SU127 | UT12 | Thailand | 0 | 0.31 | 0.68 | 3 |
| SU128 | MPT02-612 | Thai breeding line | 0 | 0 | 1 | 3 |
| SU129 | MPT14-1-172 | Thai breeding line | 0 | 1 | 0 | 2 |
| SU130 | MPT06-344 | Thai breeding line | 0 | 0.98 | 0.02 | 2 |
| SU131 | MPT14-1-976 | Thai breeding line | 0 | 0.99 | 0.01 | 2 |
| SU132 | Q115 | Australia | 1 | 0 | 0 | 1 |
| SU133 | MPT14-9-133 | Thai breeding line | 0.98 | 0 | 0.02 | 1 |
| SU134 | Co1148 | India | 0.87 | 0.08 | 0.05 | 1 |
| SU135 | MPT10-54 | Thai breeding line | 0.11 | 0.89 | 0 | 2 |
| SU136 | MPT13-118 | Thai breeding line | 0 | 0.7 | 0.3 | Admixture |
| SU137 | MPT14-5-216 | Thai breeding line | 0 | 1 | 0 | 2 |
| SU138 | NCo382 | South Africa | 0.99 | 0 | 0 | 1 |
| SU139 | TBy20-2248 | Thailand | 0 | 1 | 0 | 2 |
| SU140 | CP61-39 | USA | 0.06 | 0.93 | 0.02 | 2 |
| SU141 | MP3 | Thai breeding line | 0 | 1 | 0 | 2 |
| SU142 | MPT14-1-468 | Thai breeding line | 0 | 1 | 0 | 2 |
| SU143 | IAC49-131 | Brazil | 1 | 0 | 0 | 1 |
| SU144 | MPT11-181 | Thai breeding line | 0.01 | 0.25 | 0.74 | 3 |
| SU145 | ROC22 | Taiwan | 0.97 | 0.02 | 0.01 | 1 |
| SU146 | K88-92 | Thailand | 0 | 0 | 1 | 3 |
| SU147 | KKU99-01 | Thailand | 0 | 1 | 0 | 2 |
| SU148 | CAC57-23 | Philippines | 1 | 0 | 0 | 1 |
| SU149 | CP75-330 | USA | 0.97 | 0.03 | 0.01 | 1 |
| SU150 | CP110 | USA | 0 | 1 | 0 | 2 |
| SU151 | D158-41 | Guyana | 1 | 0 | 0 | 1 |
| SU152 | IAC52-156 | Brazil | 1 | 0 | 0 | 1 |
| SU153 | KKU99-03 | Thailand | 0 | 0 | 1 | 3 |
| SU154 | MPT14-1-546 | Thai breeding line | 0 | 1 | 0 | 2 |
| SU155 | MPT03-166 | Thai breeding line | 0 | 0 | 1 | 3 |
| SU156 | Biotec 6 | Thailand | 0 | 1 | 0 | 2 |
| SU157 | K84-200 | Thailand | 0 | 1 | 0 | 2 |
| SU158 | MPT04-204 | Thai breeding line | 0 | 0 | 1 | 3 |
| SU159 | TBy26-1255 | Thailand | 0 | 1 | 0 | 2 |

**Table S2.** Membership function value of drought tolerance (MFVD) for each drought tolerance category and classification of sugarcane accessions in each category

| Drought tolerance | Plant cane | Ratoon Cane |
|---|---|---|
| Highly drought tolerant (MFVD ≥ 7.0) | SU138 | |
| Drought tolerant (0.6 ≤ MFVD < 0.7) | SU025, SU108, SU156 | SU005, SU036, SU055, SU121, SU126, SU144, SU154, SU158 |
| Moderate drought tolerant (0.4 ≤ MFVD <0.6) | SU005, SU007, SU009, SU013, SU021, SU024, SU026, SU027, SU031, SU038, SU039, SU040, SU044, SU046, SU049, SU050, SU053, SU054, SU055, SU061, SU062, SU064, SU066, SU067, SU071, SU075, SU078, SU079, SU082, SU083, SU087, SU088, SU093, SU096, SU100, SU101, SU103, SU107, SU110, SU113, SU115, SU116, SU118, SU121, SU122, SU125, SU126, SU129, SU130, SU131, SU135, SU136, SU137, SU140, SU141, SU143, SU144, SU146, SU147, SU154, SU155, SU158 | SU002, SU006, SU007, SU009, SU025, SU031, SU034, SU037, SU039, SU040, SU042, SU053, SU056, SU059, SU061, SU064, SU072, SU077, SU078, SU087, SU088, SU090, SU092, SU093, SU094, SU096, SU108, SU110, SU114, SU115, SU116, SU128, SU135, SU136, SU138, SU140, SU155, SU156, SU157 |
| Highly drought susceptible (MFVD < 0.2) | SU002, SU014, SU015, SU016, SU028, SU032, SU043, SU068, SU074, SU080, SU117, SU142, SU152, SU153 | SU003, SU010, SU011, SU012, SU014, SU015, SU016, SU017, SU021, SU028, SU032, SU033, SU041, SU043, SU046, SU048, SU051, SU052, SU054, SU057, SU058, SU060, SU062, SU067, SU068, SU071, SU074, SU075, SU080, SU081, SU082, SU083, SU084, SU086, SU089, SU091, SU100, SU102, SU104, SU105, SU109, SU117, SU127, SU132, SU133, SU139, SU150, SU151, SU152, SU153 |

**Table S3.** Groups of candidate genes selected for SNP/InDel discovery

| Group | Function | Number of genes | Reference |
|---|---|---|---|
| Physiological adaptation | Water and ion movement<br>Chaperone functions<br>Osmotic homeostasis<br>Detoxification | 117 | [78-80] |
| Molecular adaptation | Protection factor<br>Channels and transporters<br>Regulatory proteins<br>Signal transduction | | |
| Phytohormone metabolism | Auxin metabolism<br>ABA metabolism<br>Ethylene metabolism | 183 | [81] |
| Drought inducible genes | Gene expression data | 349 | [11,82,83] |

**Table S4.** Statistical summary of SNPs/InDels markers in 649 targeted genes

| Chromosome | Genes | Probes | Coverage (bp) | Raw variants | | Biallelic variants | | Remove missing data (10%) | Remove rare variants (MAF < 5%) | Remove variants with LD | Markers for association study | | Marker / gene |
|---|---|---|---|---|---|---|---|---|---|---|---|---|---|
| | | | | SNP | InDel | SNP | InDel | | | | SNP | InDel | |
| 1 | 133 | 2,617 | 255,210 | 34,477 | 8,096 | 33,455 | 7,150 | 30,893 | 5,901 | 2,030 | 1,863 | 167 | 15.26 |
| 2 | 79 | 1,587 | 155,070 | 22,817 | 4,992 | 22,122 | 4,330 | 20,126 | 4,048 | 1,386 | 1,282 | 104 | 17.54 |
| 3 | 99 | 1,913 | 188,460 | 26,256 | 6,145 | 25,496 | 5,435 | 23,882 | 4,068 | 1,574 | 1,425 | 149 | 15.90 |
| 4 | 74 | 1,304 | 128,130 | 19,832 | 4,585 | 19,151 | 4,044 | 18,274 | 3,182 | 1,180 | 1,085 | 95 | 15.95 |
| 5 | 18 | 375 | 37,320 | 7,431 | 1,836 | 7,069 | 1,602 | 6,602 | 1,387 | 471 | 441 | 30 | 26.17 |
| 6 | 65 | 1,003 | 98,670 | 16,732 | 3,786 | 16,215 | 3,342 | 14,157 | 2,705 | 1,003 | 929 | 74 | 15.43 |
| 7 | 43 | 755 | 73,830 | 13,058 | 2,911 | 12,607 | 2,574 | 11,884 | 2,333 | 915 | 876 | 39 | 21.28 |
| 8 | 21 | 385 | 37,980 | 7,221 | 1,408 | 6,966 | 1,236 | 5,915 | 1,143 | 405 | 380 | 25 | 19.29 |
| 9 | 57 | 963 | 94,260 | 15,952 | 3,581 | 15,349 | 3,173 | 14,297 | 2,885 | 1,254 | 1,189 | 65 | 22.00 |
| 10 | 60 | 1,411 | 138,000 | 23,268 | 5,494 | 22,333 | 4,817 | 22,278 | 4,112 | 1,515 | 1,351 | 164 | 25.25 |
| All | 649 | 12,313 | 1,206,930 | 187,044 | 42,834 | 180,763 | 37,703 | 168,308 | 31,764 | 11,733 | 10,821 | 912 | 18.19 |

**Table S5.** SNPs/InDels markers significantly associated with yield-related traits, sugar-related traits, and drought tolerance indices under non-stressed (NS) and water-stressed (WS) conditions in plant cane (P) and ratoon cane (R). Significant marker-trait association based on Bonferroni correction with $\alpha$=0.05 (p value ≤ $4.26\times10^{-6}$), CL = cane length, CD = cane diameter, IL = internode length, SCW = single cane weight, NMC = number of millable canes, CY = cane yield, CCS = commercial cane sugar, FB = fiber, BR = brix, PO = polarization, PU = purity, SY = sugar yield, RA = ratooning ability; DC = Drought-tolerant coefficient of each yield traits; MFVD = the average value of the membership function of all the traits

| Trait | Marker | Condition (season) | Chromosome / Position | *p*-value | PVE (%) | Gene | Description |
|---|---|---|---|---|---|---|---|
| *Physiological and molecular adaptation* | | | | | | | |
| CL | SNP11382 | NS (P) | 3 / 6707337 | 2.97E-06 | 12.90 | Sh03_g003750 | Putative prolyl 4-hydroxylase 7 |
| | SNP13827 | NS (P) | 3 / 43166999 | 3.42E-06 | 12.75 | Sh03_g025700 | Heat shock cognate 70 kDa protein |
| | SNP06991 | NS (R) | 2 / 7100211 | 6.16E-09 | 16.72 | Sh02_g004300 | Similar to Os07g0194500 protein |
| CD | SNP14174 | NS (P) | 3 / 47197032 | 4.22E-07 | 10.57 | Sh03_g028350 | Lipoxygenase |
| | SNP23365 | NS (R) | 7 / 6153731 | 1.76E-11 | 21.43 | Sh07_g004490 | Protein LSD1 |
| | SNP27023 | WS (P) | 9 / 4502081 | 2.03E-07 | 12.67 | Sh09_g002860 | Sugar transporter ERD6-like 14 |
| | SNP30896 | WS (P) | 10 / 10317485 | 1.77E-10 | 19.98 | Sh10_g006580 | Pectinesterase |
| | SNP26624 | WS (R) | 8 / 24228458 | 2.68E-06 | 9.96 | Sh08_g013050 | Sodium/hydrogen exchanger 7 |
| | SNP30132 | WS (R) | 10 / 2948611 | 1.12E-07 | 12.54 | Sh10_g001870 | Pathogenesis-related protein PRMS |
| SCW | SNP27647 | NS (R) | 9 / 7169040 | 4.30E-07 | 11.53 | Sh09_g004400 | Similar to Os05g0155200 protein |
| | SNP26148 | WS (P) | 8 / 16789699 | 3.24E-06 | 12.84 | Sh08_g008700 | Heat shock cognate 70 kDa protein 2 |
| RA | InDel02945 | NS (R) | 9 / 25713932 | 8.23E-06 | 11.82 | Sh09_g013120 | Nuclear transcription factor Y subunit B |
| SY | SNP12296 | WS (P) | 3 / 18382076 | 3.31E-08 | 13.72 | Sh03_g010910 | Neutral/alkaline invertase 1, mitochondrial |
| CCS | SNP06940 | NS (P) | 2 / 7066535 | 2.22E-07 | 12.47 | Sh02_g004260 | Prolyl 4-hydroxylase 2 |
| | SNP26779 | WS (R) | 9 / 3288413 | 4.35E-07 | 12.04 | Sh09_g001950 | Similar to Os05g0120200 protein |
| PO | SNP19459 | NS (P) | 5 / 22469127 | 1.17E-07 | 12.95 | Sh05_g011820 | Linoleate 9S-lipoxygenase 2 |
| | SNP04788 | NS (R) | 1 / 52186011 | 5.57E-09 | 16.32 | Sh01_g031140 | Hexosyltransferase |
| | InDel01376 | NS (R) | 3 / 34660924 | 6.36E-07 | 11.28 | Sh03_g019550 | Transcription factor RAX3 |
| | SNP26801 | WS (P) | 9 / 3288740 | 3.14E-06 | 10.18 | Sh09_g001950 | Similar to Os05g0120200 protein |
| PU | SNP06940 | NS (P) | 2 / 7066535 | 8.95E-07 | 10.59 | Sh02_g004260 | Prolyl 4-hydroxylase 2 |
| | InDel01332 | WS (P) | 3 / 30050757 | 1.16E-07 | 12.34 | Sh03_g016850 | Similar to ABRE-binding factor BZ-1 |
| FB | SNP22244 | NS (P) | 6 / 28323255 | 5.37E-09 | 15.55 | Sh06_g016150 | Chloroplast lipocalin |
| | SNP02972 | NS (R) | 1 / 39909160 | 1.52E-06 | 13.66 | Sh01_g023060 | Similar to Proline dehydrogenase family protein |
| | SNP24360 | WS (P) | 7 / 19145418 | 1.30E-06 | 10.32 | Sh07_g010850 | Similar to Os08g0451800 protein |
| $DC_{IL}$ | SNP16318 | Index (P) | 4 / 26592249 | 1.03E-07 | 12.48 | Sh04_g015090 | Transcription factor MYB62 |
| $DC_{SCW}$ | SNP08202 | Index (P) | 2 / 27902871 | 1.54E-07 | 12.94 | Sh02_g014580 | BZIP transcription factor |
| | SNP01278 | Index (R) | 1 / 11528786 | 2.06E-09 | 17.79 | Sh01_g008050 | Acc synthase |
| | SNP10036 | Index (R) | 2 / 44603116 | 1.15E-08 | 15.66 | Sh02_g025960 | Polyol transporter 5 |
| $DC_{CY}$ | SNP01310 | Index (P) | 1 / 11529997 | 3.85E-08 | 13.98 | Sh01_g008050 | Acc synthase |
| | SNP16972 | Index (P) | 4 / 37082704 | 1.16E-06 | 11.05 | Sh04_g021630 | Trehalose 6-phosphate phosphatase |
| | SNP32955 | Index (R) | 10 / 27726604 | 1.96E-08 | 14.46 | Sh10_g014930 | Gl1 protein |

| Trait | Marker | Condition (season) | Chromosome / Position | P value | PVE (%) | Gene | Description |
|---|---|---|---|---|---|---|---|
| *Physiological and molecular adaptation* | | | | | | | |
| $DC_{SY}$ | SNP01310 | Index (P) | 1 / 11529997 | 4.99E-09 | 16.39 | Sh01_g008050 | Acc synthase |
| | InDel00906 | Index (P) | 2 / 31511091 | 9.57E-08 | 13.47 | Sh02_g016830 | Similar to Os09g0499000 protein |
| | SNP14149 | Index (P) | 3 / 47196416 | 3.12E-06 | 9.85 | Sh03_g028350 | Lipoxygenase |
| *Phytohormone metabolism* | | | | | | | |
| CL | SNP21180 | NS (R) | 6 / 15936064 | 7.76E-09 | 15.85 | Sh06_g008570 | Cytochrome b561 and DOMON domain-containing protein |
| | SNP30086 | NS (R) | 10 / 1993219 | 6.17E-08 | 13.99 | Sh10_g001250 | UDP-glucose: 2-hydroxyflavanone C-glucosyltransferase |
| | SNP05708 | WS (R) | 1 / 63992266 | 2.25E-06 | 9.24 | Sh01_g038920 | Os03g0194350 protein |
| | SNP21283 | WS (R) | 6 / 16722810 | 7.00E-08 | 12.20 | Sh06_g009100 | Similar to OSIGBa0145C12.1 protein |
| | SNP25333 | WS (R) | 7 / 27924992 | 4.28E-07 | 11.44 | Sh07_g016790 | Ethylene insensitive 3-like 3 protein |
| CD | SNP15778 | NS (R) | 4 / 9688810 | 4.94E-08 | 12.74 | Sh04_g006500 | EIN3-binding F-box protein 1 |
| | SNP02074 | WS (P) | 1 / 18852079 | 2.55E-07 | 12.15 | Sh01_g012390 | 9-cis-epoxycarotenoid dioxygenase 1, chloroplastic |
| | SNP28798 | WS (P) | 9 / 20675137 | 2.41E-07 | 11.82 | Sh09_g010250 | Glycosyltransferase |
| | SNP29315 | WS (P) | 9 / 30148431 | 4.13E-06 | 8.92 | Sh09_g016200 | Protein continuous vascular ring 1 |
| IL | SNP11371 | NS (R) | 3 / 6123382 | 1.71E-07 | 13.04 | Sh03_g003440 | Transaminase/ transferase isoform 1 |
| SCW | SNP28798 | NS (P) | 9 / 20675137 | 8.81E-08 | 13.41 | Sh09_g010250 | Glycosyltransferase |
| | SNP31040 | NS (R) | 10 / 12574379 | 8.07E-08 | 13.76 | Sh10_g007750 | Similar to plant protein family protein, expressed |
| | SNP28798 | WS (P) | 9 / 20675137 | 1.60E-06 | 13.59 | Sh09_g010250 | Glycosyltransferase |
| NMC | SNP28760 | NS (P) | 9 / 20674823 | 2.17E-09 | 17.51 | Sh09_g010250 | Glycosyltransferase |
| | SNP05000 | NS (R) | 1 / 54088475 | 3.57E-08 | 12.98 | Sh01_g032260 | Abscisic acid receptor PYL5 |
| | SNP28810 | NS (R) | 9 / 23085243 | 1.22E-06 | 9.75 | Sh09_g011670 | Serine/threonine-protein kinase SAPK4 |
| | SNP17032 | WS (P) | 4 / 38314918 | 3.57E-06 | 12.82 | Sh04_g022390 | Auxin-responsive protein SAUR32 |
| CY | SNP00918 | NS (P) | 1 / 5872959 | 3.69E-06 | 12.67 | Sh01_g004020 | Indole-3-acetaldehyde oxidase |
| | SNP02074 | NS (P) | 1 / 18852079 | 3.88E-06 | 12.62 | Sh01_g012390 | 9-cis-epoxycarotenoid dioxygenase 1, chloroplastic |
| SY | SNP20355 | NS (P) | 6 / 6883524 | 9.92E-07 | 9.96 | Sh06_g003010 | Ethylene-responsive transcription factor ERF113 |
| | SNP20008 | NS (R) | 6 / 4990510 | 1.26E-06 | 10.64 | Sh06_g002150 | Momilactone A synthase |
| | SNP11186 | WS (P) | 3 / 5339732 | 3.10E-06 | 9.22 | Sh03_g002910 | 1-aminocyclopropane-1-carboxylate oxidase |
| | SNP17009 | WS (P) | 4 / 38313956 | 7.59E-10 | 18.56 | Sh04_g022390 | Auxin-responsive protein SAUR32 |
| BR | SNP28774 | NS (R) | 9 / 20674923 | 1.44E-06 | 10.68 | Sh09_g010250 | Glycosyltransferase |
| CCS | SNP17531 | NS (P) | 4 / 39831899 | 5.41E-08 | 12.77 | Sh04_g023500 | Indole-3-acetic acid-induced protein ARG7 |
| | SNP20342 | NS (R) | 6 / 6883094 | 2.09E-06 | 10.00 | Sh06_g003010 | Ethylene-responsive transcription factor ERF113 |
| | InDel02259 | NS (R) | 6 / 22581038 | 2.59E-06 | 9.31 | Sh06_g012530 | Auxin-independent growth promoter |
| | SNP28774 | NS (R) | 9 / 20674923 | 3.67E-07 | 11.96 | Sh09_g010250 | Glycosyltransferase |
| | SNP23693 | WS (P) | 7 / 14061956 | 2.92E-07 | 12.21 | Sh07_g008590 | Putative protein |
| | SNP03745 | WS (R) | 1 / 43409921 | 3.60E-06 | 9.48 | Sh01_g025450 | GH31 |
| | SNP10730 | WS (R) | 3 / 1588896 | 4.59E-08 | 13.97 | Sh03_g000970 | Similar to Ethylene insensitive 2 |
| PO | SNP12207 | NS (P) | 3 / 17592105 | 2.01E-06 | 9.75 | Sh03_g010510 | Ethylene-responsive transcription factor RAP2-3 |

| Trait | Marker | Condition (season) | Chromosome / Position | P value | PVE (%) | Gene | Description |
|---|---|---|---|---|---|---|---|
| *Phytohormone metabolism* | | | | | | | |
| PO | SNP17531 | NS (P) | 4 / 39831899 | 2.26E-06 | 9.94 | Sh04_g023500 | Indole-3-acetic acid-induced protein ARG7 |
| | SNP23693 | NS (R) | 7 / 14061956 | 1.28E-06 | 10.47 | Sh07_g008590 | Putative protein |
| | SNP00861 | WS (P) | 1 / 5871494 | 1.51E-06 | 10.05 | Sh01_g004020 | Indole-3-acetaldehyde oxidase |
| | SNP13002 | WS (R) | 3 / 35125167 | 2.05E-06 | 10.68 | Sh03_g020000 | GDSL esterase/lipase |
| PU | SNP00861 | NS (P) | 1 / 5871494 | 2.88E-06 | 10.06 | Sh01_g004020 | Indole-3-acetaldehyde oxidase |
| | SNP17393 | NS (R) | 4 / 38881710 | 3.60E-06 | 9.60 | Sh04_g022760 | Similar to Chromosome chr18 scaffold_1 |
| | SNP19425 | NS (R) | 5 / 17948281 | 1.25E-06 | 10.45 | Sh05_g009110 | HVA22-like protein |
| | SNP21656 | NS (R) | 6 / 21293971 | 8.56E-09 | 14.48 | Sh06_g011620 | EREBP-4 like protein |
| | SNP23693 | NS (R) | 7 / 14061956 | 1.08E-11 | 20.69 | Sh07_g008590 | Putative protein |
| | InDel02232 | WS (P) | 6 / 21259097 | 1.40E-06 | 10.53 | Sh06_g011580 | Ethylene-responsive transcription factor 5 |
| FB | SNP16699 | NS (P) | 4 / 33551613 | 2.60E-06 | 10.09 | Sh04_g019510 | Pollen-specific leucine-rich repeat extensin-like protein 4 |
| | SNP32494 | NS (P) | 10 / 22150546 | 1.55E-06 | 10.66 | Sh10_g011820 | EREBP-4 like protein |
| | SNP19005 | WS (P) | 5 / 10175848 | 1.64E-08 | 15.51 | Sh05_g005730 | Flavin-containing monooxygenase |
| | SNP32494 | WS (P) | 10 / 22150546 | 1.29E-11 | 22.38 | Sh10_g011820 | EREBP-4 like protein |
| $DC_{SCW}$ | SNP02600 | Index (R) | 1 / 31339306 | 1.51E-07 | 13.25 | Sh01_g019210 | Perakine reductase |
| | SNP05708 | Index (R) | 1 / 63992266 | 1.06E-06 | 11.13 | Sh01_g038920 | Os03g0194350 protein |
| $DC_{CY}$ | InDel03451 | Index (P) | 10 / 28410796 | 1.45E-09 | 17.59 | Sh10_g015190 | Auxin efflux carrier component |
| | SNP00861 | Index (R) | 1 / 5871494 | 1.32E-11 | 22.51 | Sh01_g004020 | Indole-3-acetaldehyde oxidase |
| | SNP19400 | Index (R) | 5 / 17947231 | 1.14E-07 | 10.88 | Sh05_g009110 | HVA22-like protein |
| $DC_{SY}$ | SNP15547 | Index (P) | 4 / 5566534 | 1.27E-06 | 11.46 | Sh04_g003750 | Cullin-associated NEDD8-dissociated protein 1 |
| | SNP17009 | Index (P) | 4 / 38313956 | 1.66E-06 | 10.35 | Sh04_g022390 | Auxin-responsive protein SAUR32 |
| | SNP17032 | Index (P) | 4 / 38314918 | 3.94E-08 | 14.79 | Sh04_g022390 | Auxin-responsive protein SAUR32 |
| | SNP28780 | Index (P) | 9 / 20674967 | 6.95E-09 | 14.77 | Sh09_g010250 | Glycosyltransferase |
| | SNP14932 | Index (R) | 3 / 54184437 | 2.53E-11 | 22.46 | Sh03_g033410 | Putative protein |
| MFVD | SNP17032 | Index (P) | 4 / 38314918 | 3.11E-06 | 9.66 | Sh04_g022390 | Auxin-responsive protein SAUR32 |
| | SNP16162 | Index (R) | 4 / 21724210 | 1.02E-06 | 10.26 | Sh04_g012100 | Ethylene-responsive transcription factor ERF021 |
| | SNP21960 | Index (R) | 6 / 22584823 | 2.15E-09 | 15.74 | Sh06_g012530 | Auxin-independent growth promoter |
| *Drought inducible genes* | | | | | | | |
| CL | SNP24870 | NS (P) | 7 / 23832165 | 4.25E-06 | 12.52 | Sh07_g013900 | ARO1-like protein 1 |
| | SNP03332 | NS (R) | 1 / 41297435 | 1.08E-06 | 10.72 | Sh01_g023980 | Peroxidase |
| | SNP15892 | NS (R) | 4 / 15583047 | 1.41E-07 | 12.30 | Sh04_g009360 | Peroxisomal fatty acid beta-oxidation multifunctional protein |
| | SNP15637 | WS (R) | 4 / 5794042 | 1.38E-07 | 12.31 | Sh04_g003900 | Phosphoglycerate kinase, cytosolic |
| | SNP18377 | WS (R) | 5 / 1856393 | 2.05E-06 | 10.31 | Sh05_g001300 | Protein Zinc induced facilitator-like 1 |
| CD | SNP05953 | NS (P) | 1 / 66130495 | 8.25E-08 | 13.07 | Sh01_g040220 | Dof-type zinc finger protein |
| | SNP22840 | NS (P) | 6 / 33828886 | 1.00E-06 | 10.59 | Sh06_g020100 | DNA mismatch repair protein MSH3 |
| | SNP24679 | NS (P) | 7 / 22355846 | 1.15E-08 | 15.29 | Sh07_g012680 | Conserved hypothetical protein |
| | SNP05953 | NS (R) | 1 / 66130495 | 1.70E-12 | 24.02 | Sh01_g040220 | Dof-type zinc finger protein |

| Trait | Marker | Condition (season) | Chromosome / Position | P value | PVE (%) | Gene | Description |
|---|---|---|---|---|---|---|---|
| *Drought inducible genes* | | | | | | | |
| CD | SNP13091 | NS (R) | 3 / 35520399 | 2.43E-08 | 14.38 | Sh03_g020240 | Reticulon-like protein |
| | SNP07895 | WS (R) | 2 / 19935393 | 2.26E-09 | 16.41 | Sh02_g010540 | Similar to Apospory-associated protein C-like |
| | SNP15050 | WS (R) | 4 / 1321819 | 2.22E-06 | 9.81 | Sh04_g000730 | Glutamate receptor |
| | SNP17340 | WS (R) | 4 / 38804056 | 1.12E-07 | 11.74 | Sh04_g022680 | Salutaridine reductase |
| | SNP24679 | WS (R) | 7 / 22355846 | 1.59E-06 | 10.60 | Sh07_g012680 | Conserved hypothetical protein |
| | SNP33171 | WS (R) | 10 / 29027260 | 4.87E-07 | 10.72 | Sh10_g015660 | Polyubiquitin 4 |
| IL | SNP15240 | NS (R) | 4 / 1972364 | 9.70E-07 | 10.85 | Sh04_g001400 | SKP1-like protein 11 |
| | InDel03314 | NS (R) | 10 / 21921494 | 9.00E-07 | 10.91 | Sh10_g011740 | Non-specific serine/threonine protein kinase |
| | SNP19725 | WS (P) | 6 / 1127030 | 1.72E-06 | 13.56 | Sh06_g000770 | Peptidyl-prolyl cis-trans isomerase Pin1 |
| | InDel03511 | WS (P) | 10 / 31606658 | 1.63E-06 | 13.62 | Sh10_g017200 | Phosphatase DCR2 |
| | SNP13635 | WS (R) | 3 / 41158322 | 3.24E-06 | 13.15 | Sh03_g024540 | NADH-cytochrome b5 reductase |
| SCW | SNP13445 | NS (P) | 3 / 38125702 | 1.38E-08 | 15.04 | Sh03_g022130 | Similar to Os01g0769000 protein |
| | SNP28417 | NS (P) | 9 / 18388955 | 6.24E-08 | 12.68 | Sh09_g009130 | Lichenase-2 |
| | SNP02866 | NS (R) | 1 / 32991900 | 2.77E-08 | 14.93 | Sh01_g020090 | Cyclic dof factor 1 |
| | SNP04329 | NS (R) | 1 / 47872373 | 6.12E-08 | 13.37 | Sh01_g028210 | Similar to Expressed protein |
| | SNP05953 | NS (R) | 1 / 66130495 | 3.38E-06 | 9.63 | Sh01_g040220 | Dof-type zinc finger protein |
| | SNP25508 | NS (R) | 8 / 301065 | 7.76E-08 | 12.92 | Sh08_g000220 | E3 SUMO-protein ligase SIZ2 |
| | SNP25644 | NS (R) | 8 / 5768339 | 3.20E-07 | 12.27 | Sh08_g003660 | Tubby-like F-box protein |
| | SNP02285 | WS (P) | 1 / 24019722 | 3.25E-06 | 12.84 | Sh01_g015370 | Serine incorporator 3 |
| NMC | SNP05953 | NS (P) | 1 / 66130495 | 3.21E-07 | 10.86 | Sh01_g040220 | Dof-type zinc finger protein |
| | SNP23501 | NS (P) | 7 / 8089083 | 4.59E-07 | 12.18 | Sh07_g005700 | Aconitate hydratase |
| | SNP28844 | NS (P) | 9 / 24884959 | 9.20E-07 | 9.85 | Sh09_g012650 | Protein phosphatase 2C 3 |
| | SNP32524 | NS (P) | 10 / 22470080 | 1.48E-06 | 9.73 | Sh10_g011920 | Diacylglycerol O-acyltransferase 1-2 |
| | SNP34157 | NS (P) | 10 / 34290570 | 3.80E-08 | 13.65 | Sh10_g019050 | Phytoene synthase, chloroplastic |
| | SNP12358 | NS (R) | 3 / 22267073 | 8.89E-07 | 9.54 | Sh03_g012720 | 70 kDa peptidyl-prolyl isomerase |
| | SNP16114 | NS (R) | 4 / 18806196 | 2.38E-06 | 9.39 | Sh04_g010560 | Acetolactate synthase 1, chloroplastic |
| | SNP24461 | NS (R) | 7 / 20853629 | 1.11E-06 | 11.26 | Sh07_g011750 | Similar to Putative SEC23 |
| | InDel03500 | NS (R) | 10 / 31253769 | 1.02E-08 | 16.13 | Sh10_g016930 | Xyloglucan endotransglucosylase/hydrolase |
| SY | SNP08052 | NS (P) | 2 / 26576643 | 1.49E-08 | 14.96 | Sh02_g013850 | Similar to Os09g0439000 protein |
| | SNP27968 | NS (P) | 9 / 9561800 | 3.37E-09 | 16.33 | Sh09_g005710 | 60S ribosomal protein L13 |
| | SNP07858 | NS (R) | 2 / 19603616 | 2.21E-10 | 19.65 | Sh02_g010430 | Plant cysteine oxidase 5 |
| | SNP25403 | NS (R) | 8 / 297131 | 1.41E-06 | 10.65 | Sh08_g000220 | E3 SUMO-protein ligase SIZ2 |
| | SNP32155 | NS (R) | 10 / 20160298 | 8.03E-09 | 15.03 | Sh10_g011140 | Conserved hypothetical protein |

| Trait | Marker | Condition (season) | Chromosome / Position | P value | PVE (%) | Gene | Description |
|---|---|---|---|---|---|---|---|
| *Drought inducible genes* | | | | | | | |
| SY | InDel01261 | WS (P) | 3 / 10040814 | 2.75E-08 | 14.93 | Sh03_g005970 | E3 ubiquitin-protein ligase |
| | SNP28936 | WS (P) | 9 / 25705204 | 2.20E-06 | 9.35 | Sh09_g013110 | Putative protein |
| CCS | SNP18684 | NS (P) | 5 / 5126869 | 6.44E-07 | 10.94 | Sh05_g003160 | Long chain acyl-CoA synthetase 8 |
| | SNP21819 | NS (P) | 6 / 22119189 | 3.94E-06 | 9.72 | Sh06_g012220 | Putative similar to Expressed protein |
| | SNP25403 | NS (P) | 8 / 297131 | 1.15E-06 | 10.73 | Sh08_g000220 | E3 SUMO-protein ligase SIZ2 |
| | SNP30775 | NS (P) | 10 / 8356963 | 2.69E-06 | 10.80 | Sh10_g005390 | Cystathionine gamma-synthase |
| | SNP21122 | NS (R) | 6 / 14419780 | 3.45E-08 | 13.79 | Sh06_g007680 | Putative ADP-ribosylation factor 1 |
| | SNP08601 | WS (P) | 2 / 30539439 | 2.63E-06 | 10.08 | Sh02_g016000 | F-box domain containing protein |
| | SNP27250 | WS (P) | 9 / 4917629 | 3.16E-06 | 8.44 | Sh09_g003050 | S-adenosylmethionine synthase 1 |
| | SNP28553 | WS (P) | 9 / 20200509 | 1.53E-06 | 10.72 | Sh09_g009960 | DUF21 domain-containing protein |
| | SNP13888 | WS (R) | 3 / 44183889 | 2.13E-08 | 14.50 | Sh03_g026400 | Similar to Os01g0849500 protein |
| BR | SNP17282 | NS (P) | 4 / 38773420 | 4.34E-07 | 11.88 | Sh04_g022670 | Salutaridine reductase |
| | SNP25403 | NS (P) | 8 / 297131 | 1.55E-06 | 10.58 | Sh08_g000220 | E3 SUMO-protein ligase SIZ2 |
| | SNP29415 | NS (P) | 9 / 30947902 | 1.53E-06 | 10.27 | Sh09_g016850 | Beta-galactosidase 8 |
| | SNP08601 | NS (R) | 2 / 30539439 | 2.13E-08 | 14.42 | Sh02_g016000 | F-box domain containing protein |
| | SNP11963 | NS (R) | 3 / 14056404 | 4.31E-07 | 11.37 | Sh03_g008520 | RNA binding protein |
| | SNP22843 | NS (R) | 6 / 33828914 | 2.84E-06 | 10.24 | Sh06_g020100 | DNA mismatch repair protein MSH3 |
| | InDel00869 | WS (P) | 2 / 28754538 | 1.89E-08 | 12.85 | Sh02_g015090 | Similar to Os02g0781700 protein |
| | SNP12411 | WS (P) | 3/ 22271193 | 6.26E-09 | 13.98 | Sh03_g012720 | 70 kDa peptidyl-prolyl isomerase |
| | SNP16838 | WS (P) | 4 / 34252556 | 3.66E-06 | 8.20 | Sh04_g020080 | Myb-related protein Zm1 |
| | SNP22843 | WS (P) | 6 / 33828914 | 8.18E-09 | 15.53 | Sh06_g020100 | DNA mismatch repair protein MSH3 |
| | SNP31664 | WS (P) | 10 / 16011797 | 3.22E-06 | 9.09 | Sh10_g009590 | Jumonji transcription factor |
| | SNP04603 | WS (R) | 1 / 49414358 | 2.80E-06 | 13.26 | Sh01_g029210 | Similar to Importin subunit alpha-2 |
| PO | SNP08596 | NS (P) | 2 / 30539289 | 4.09E-08 | 12.00 | Sh02_g016000 | F-box domain containing protein |
| | SNP08829 | NS (P) | 2 / 31960537 | 1.17E-07 | 13.16 | Sh02_g017140 | Ubiquitinyl hydrolase 1 |
| | SNP10174 | NS (P) | 2 / 45553777 | 6.09E-07 | 10.67 | Sh02_g026710 | Conserved hypothetical protein |
| | SNP27268 | NS (P) | 9 / 4917949 | 1.80E-10 | 17.76 | Sh09_g003050 | S-adenosylmethionine synthase 1 |
| | SNP17462 | WS (P) | 4 / 39449984 | 7.36E-08 | 12.93 | Sh04_g023190 | Ferredoxin--nitrite reductase, chloroplastic |
| | InDel02088 | WS (P) | 6 / 1309716 | 1.26E-06 | 10.44 | Sh06_g000950 | Pyruvate dehydrogenase E1 component subunit alpha-3, chloroplastic |
| | SNP13100 | WS (R) | 3 / 35520862 | 8.90E-07 | 10.03 | Sh03_g020240 | Reticulon-like protein |
| | SNP14699 | WS (R) | 3 / 53402844 | 2.55E-06 | 10.51 | Sh03_g032790 | Similar to Putative beta-galactosidase |
| | SNP31150 | WS (R) | 10 / 12672682 | 5.29E-07 | 11.66 | Sh10_g007850 | Protein exordium |

| Trait | Marker | Condition (season) | Chromosome / Position | P value | PVE (%) | Gene | Description |
|---|---|---|---|---|---|---|---|
| *Drought inducible genes* | | | | | | | |
| PU | SNP15639 | NS (P) | 4 / 5794101 | 1.16E-08 | 15.22 | Sh04_g003900 | Phosphoglycerate kinase, cytosolic |
| | SNP10544 | NS (R) | 2 / 52479176 | 4.41E-10 | 17.82 | Sh02_g031600 | 26S protease regulatory subunit 4 homolog |
| | SNP13643 | NS (R) | 3 / 41158911 | 5.76E-09 | 14.63 | Sh03_g024540 | NADH-cytochrome b5 reductase |
| | SNP29239 | NS (R) | 9 / 28581507 | 1.64E-07 | 11.89 | Sh09_g015130 | Similar to Chromosome chr14 scaffold_21 |
| | SNP24014 | WS (P) | 7 / 16722012 | 7.01E-09 | 14.86 | Sh07_g009680 | Similar to FYVE finger-containing phosphoinositide kinase-like |
| | SNP24669 | WS (P) | 7 / 22355558 | 7.45E-07 | 11.34 | Sh07_g012680 | Conserved hypothetical protein |
| | SNP26435 | WS (P) | 8 / 23612728 | 1.68E-06 | 10.57 | Sh08_g012730 | Conserved hypothetical protein |
| FB | SNP01128 | NS (P) | 1 / 10267445 | 4.13E-06 | 7.93 | Sh01_g007080 | ATP sulfurylase |
| | SNP16094 | NS (P) | 4 / 18805244 | 1.73E-08 | 14.85 | Sh04_g010560 | Acetolactate synthase 1, chloroplastic |
| | SNP04871 | NS (R) | 1 / 52787966 | 1.60E-06 | 13.60 | Sh01_g031440 | Phytochrome B |
| | SNP22770 | WS (P) | 6 / 32768250 | 1.89E-08 | 15.04 | Sh06_g019340 | Anamorsin homolog |
| | SNP29592 | WS (P) | 9 / 33106253 | 1.01E-07 | 11.91 | Sh09_g018430 | Chlorophyll a-b binding protein M9, chloroplastic |
| | InDel03129 | WS (P) | 10 / 6354878 | 2.89E-06 | 10.29 | Sh10_g004040 | Putative 5-methyltetrahydropteroyltriglutamate-homocysteine methyltransferase |
| $DC_{IL}$ | InDel03511 | Index (P) | 10 / 31606658 | 5.91E-07 | 11.54 | Sh10_g017200 | Phosphatase DCR2 |
| $DC_{SCW}$ | SNP05965 | Index (P) | 1 / 66130846 | 2.02E-15 | 30.52 | Sh01_g040220 | Dof-type zinc finger protein |
| | SNP08083 | Index (R) | 2 / 26578367 | 7.93E-07 | 11.23 | Sh02_g013850 | Similar to Os09g0439000 protein |
| | SNP15105 | Index (R) | 4 / 1325019 | 3.85E-07 | 12.23 | Sh04_g000730 | Glutamate receptor |
| | SNP20588 | Index (R) | 6 / 11281177 | 7.18E-12 | 23.19 | Sh06_g005230 | Similar to OSJNBa0058G03.7 protein |
| $DC_{CY}$ | SNP25508 | Index (R) | 8 / 301065 | 6.95E-07 | 10.95 | Sh08_g000220 | E3 SUMO-protein ligase SIZ2 |
| | InDel03254 | Index (R) | 10 / 16015694 | 1.94E-06 | 10.74 | Sh10_g009590 | Jumonji transcription factor |
| $DC_{SY}$ | SNP10998 | Index (P) | 3 / 3782911 | 3.77E-07 | 12.21 | Sh03_g002230 | Similar to Expressed protein |
| | SNP27968 | Index (P) | 9 / 9561800 | 1.14E-12 | 24.96 | Sh09_g005710 | 60S ribosomal protein L13 |
| | SNP00390 | Index (R) | 1 / 3460814 | 2.61E-06 | 9.58 | Sh01_g002510 | Conserved hypothetical protein |
| | SNP03302 | Index (R) | 1 / 41296454 | 1.02E-07 | 13.50 | Sh01_g023980 | Peroxidase |
| | SNP07644 | Index (R) | 2 / 16207065 | 1.38E-06 | 10.57 | Sh02_g009160 | Beta-amylase |
| | InDel01155 | Index (R) | 3 / 3785054 | 2.23E-06 | 9.46 | Sh03_g002230 | Similar to Expressed protein |
| | SNP16114 | Index (R) | 4 / 18806196 | 6.76E-11 | 21.54 | Sh04_g010560 | Acetolactate synthase 1, chloroplastic |
| MFVD | SNP25057 | Index (P) | 7 / 26076236 | 5.75E-07 | 10.37 | Sh07_g015500 | Putative protein |
| | SNP28494 | Index (P) | 9 / 19319254 | 5.76E-07 | 10.36 | Sh09_g009570 | Ribosomal protein |
| | SNP03302 | Index (R) | 1 / 41296454 | 4.13E-07 | 11.86 | Sh01_g023980 | Peroxidase |
